# Mechanical Properties of Chemically Modified Clay

**DOI:** 10.1038/s41598-019-49972-7

**Published:** 2019-09-23

**Authors:** Marta S. S. Gusmão, Priya Gopal, Ilaria Siloi, Stefano Curtarolo, Marco Fornari, Marco Buongiorno Nardelli

**Affiliations:** 1 Department of Physics, Federal University of Amazonas, Amazonas, Brazil; 2 Department of Physics, University of North Texas, Denton, TX 76203 USA; 3 Department of Physics and Science of Advanced Material Program, Central Michigan University, Mt.Pleasant, MI 48859 USA; 4 Department of Mechanical Engineering and Materials Science, Duke University, Durham, NC 27708 USA; 5 Center for Materials Genomics, Duke University, Durham, NC 27708 USA

**Keywords:** Mechanical properties, Mechanical properties, Mechanical properties, Electronic structure, Electronic structure

## Abstract

Serpentine clay minerals are found in many geological settings. The rich diversity, both in chemical composition and crystal structure, alters the elastic behavior of clay rocks significantly, thus modifying seismic and sonic responses to shaley sequences. Computation of the elastic properties is a useful tool to characterize this diversity. In this paper we use first principles methods to compare the mechanical properties of lizardite Mg_3_(Si_2_O_5_)(OH)_4_, a polymorph of serpentine family, with the new compounds derived by substituting Mg ions with isovalent elements from different chemical groups. New compounds are first selected according to chemical and geometrical stability criteria, then full elastic tensors, bulk and shear modulii, and acoustic velocities are obtained. Overall, the new compounds have a lower anisotropy and are less resistant to mechanical deformation compared to the prototype, thus providing valuable information regarding chemical composition and mechanical properties in these systems.

## Introduction

Serpentine clay minerals are Mg-rich hydrous phyllosilicates found predominantly in the Earth’s mantle, and their physical properties have major implications in diverse area of geophysics. Valuable information on the tectonic history can be gained by analyzing the structure and elastic constants of these minerals^1^. Knowledge of velocities of sedimentary rocks is mainly used in seismic imaging, hydraulic fracturing, and drilling. Since the Earth’s interior is not homogeneous, the propagation velocity of internal seismic waves depend on the physical properties of the rocks that they cross. Measuring the elastic constants directly in fine grained materials is very difficult due to the impossibility of isolating a single grain of clay; for this reason, theoretical simulations combined with experimental measures or extrapolations are useful tools to investigate mechanical properties of these systems.

**Figure 1:**
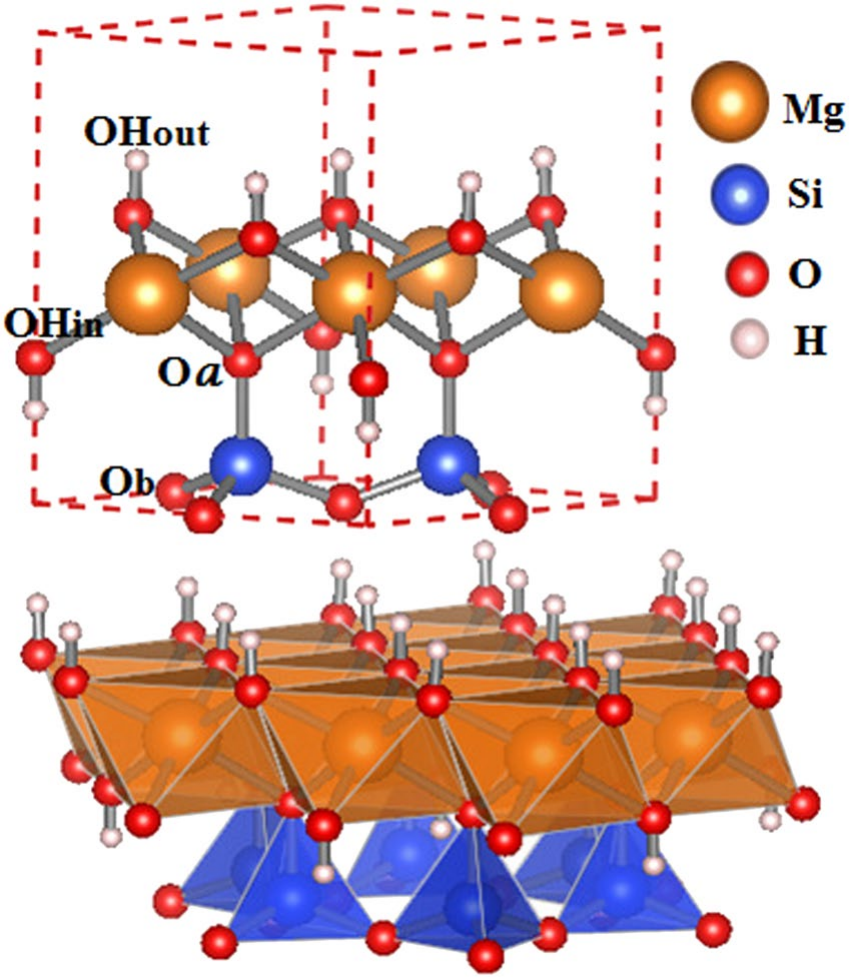
Stick-and-ball (top) and polyhedral (bottom) representation of lizardite (Mg_3_(Si_2_O_5_)(OH)_4_) crystal structure along the [001] direction. The apical and basal oxygen atoms are labeled as O_*a*_ and O_*b*_, respectively. The hydroxyl (OH)^−^ position types are also indicated. Metal cation is in the octahedral cage (orange) and Si atoms are in the tetrahedral cage (in blue).

The structures of the serpentine minerals are based on uncharged tetrahedral Si_2_(O_*b*_)_3_(O_*a*_)_2_ and octahedral Mg_3_(OH)_4_(O_*a*_)_2_ units arranged in sheets in a 1:1 ratio, where O_*a*_ and O_*b*_ are the apical and the basal oxygen atoms, respectively (Fig. 1). The serpentine group is composed of three clay minerals with the same chemical formula, but different crystal structures. The arrangement of these sheets is responsible for the different species: lizardite and antigorite (planar shape) and chrysotile (tubular form). Lizardite is Mg-rich 1:1 trioctahedral layer mineral, ideally Mg_3_(Si_2_O_5_)(OH)_4_, with space group P31m and trigonal crystal structure. The first crystal structure refinement was reported by Mellini^2^ in 1982, then other researches have performed structural studies on two polytypes of lizardite^3,4^. See Fig. 1 for the crystal structure.

A few experimental measurements on the mechanical properties have been reported by Mellini and Zanazzi^3^, Tyburczy *et al*.^5^ and Hilairet *et al*.^6^ and some theoretical studies have been done using density functional theory (DFT) calculations^7–10^. Lizardite was also readily synthesized in laboratory^11^. In addition to Mg, numerous samples of serpentine rocks contain traces of other elements like Co, Mn, Fe, Al and Zn^12^. Understanding the effect of chemical variations on the physical properties of prototype lizardite is useful in synthesis and design of new functional clay minerals and this is the focus of the present work. Here we perform a systematic first principles study of the chemical, structural and mechanical properties of isostructural new compounds derived by the chemical substitution in lizardite, where Mg in the octahedral sheet is replaced with one of the twelve elements from different chemical groups (alkaline earth metals, Be and Ca, transition metals, Ni, Mn, Fe, and Zn, post-transition metals, Al and Sn, metalloids, Ge and Te, and the nonmetal, S and Se) with the same oxidation state as Mg^2+^. We compute the full elastic tensor and the derived criteria for stability, including bulk modulus, shear modulus and acoustic velocities for the new structures and a detailed comparison is performed with the existing prototype.

The paper is organized as follows. In Sec. 1, we describe the computational method, stability criteria and key quantities used to analyze mechanical properties. In Sec. 2, we first validate the method on the prototype material, then we discuss our results on the formation energies and elastic properties of the derived systems. Sec. 3 closes the paper with a summary of the results.

## Materials and Methods

All the calculations are performed within density functional theory (DFT) using the quantum espresso (QE) code^13^ integrated in the AFLOW*π* (Automatic Flow *π*)^14,15^– a portable framework for high-throughput first principles calculations – which allows calculation of elastic properties, dielectric functions,electronic and optical properties. The crystal structures of the prototype, lizardite, was taken from the refinement reported by Mellini^2^ from American Minerologist Crystal Structure Database^16^.

After several tests (see Sec. 2), we used the revised Perdew-Burke-Ernzerhof generalized gradient approximation (PBESol)^17^ as exchange-correlation functional and ultrasoft pseudopotentials for our calculations. The kinetic energy cutoffs for the plane waves and for charge density are 60 and 600 Ry, respectively, and a 5 × 5 × 5 Monkhorst-Pack **k**-point mesh is used for all our calculations. The structures are relaxed until the convergence for forces and total energy achieve values lower than 0.1 mRy/Å, 0.01 mRy, respectively.

Lizardite has a trigonal (space group: P31m) crystal symmetry with six independent second-order elastic constants (SOEC): ${c}_{11}={c}_{22}$, *c*_12_, *c*_13_, *c*_33_ e *c*_44_ in Voigt notation (${c}_{ijkl}\to {c}_{\alpha \beta }$). We computed these values within the stress deformation approach as implemented in ElaStic code^18^ with ${c}_{ij}=\frac{\partial {\tau }_{i}}{\partial {\eta }_{j}}$, where ${\tau }_{i}$ and ${\eta }_{j}$ are the Lagrangian strain and stress, respectively. For the given space group and the Lagrangian strain values between $[\,-\,0.005,0.005]$, around 17 distorted structures are generated and each of them is then relaxed. The deformation types are defined according to Yu *et al*.^19^. We obtain the SOECs, bulk (*B*) and shear (*G*) moduli for Voigt (*V*) and Reuss (*R*) averaging procedure, Young’s modulus (*E*), Poisson’s ratio ($\nu$) and SOEC eigenvalues all of which are further used to analyze the mechanical stability of the new compounds. We use Drucker’s criteria^20,21^ for testing the mechanical stability. Additionally, the two modulii *B*_*R*_ and *G*_*R*_ values should also be positive for elastic stability. As a post-processing step, we obtain Pugh’s index^22^, *B*/*G*, and the universal elastic anisotropy index^23^ that includes all crystal symmetries,

1 $${A}^{U}=5\frac{{G}_{V}}{{G}_{R}}+\frac{{B}_{V}}{{B}_{R}}-6\ge 0,$$

also used as mechanical stability criterion for the new compounds. The compressional wave velocity, *V*_*p*_, and shear wave velocity, *V*_*s*_ are also calculated according to Birch^24^,

2 $$\rho {V}_{p}^{2}=B+\frac{4G}{3},$$

3 $$\rho {V}_{s}^{2}=G,$$

where $B=({B}_{R}+{B}_{V})/2$ and $G=({G}_{R}+{G}_{V})/2$ are the Hill-averaged bulk and shear moduli.

We also performed the calculation of frequencies of the normal vibrational modes for the prototype at the center of Brillouin zone center, $\Gamma$ point, using the PHonon code^13,25^, a part of the QE distribution.

## Results and Discussion

**Figure 2:**
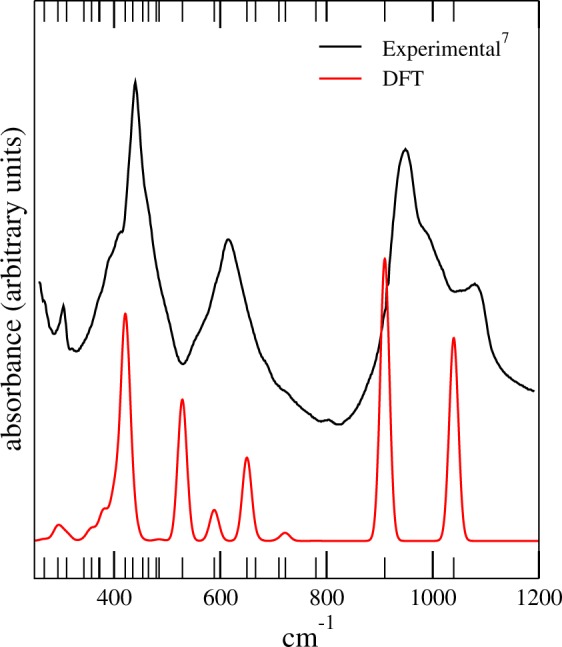
IR absorption spectra of lizardite at the center of the first Brillouin zone: Experimental (black)^7^ and theoretical (red).

In order to validate our computational approach, we perform initial tests on lizardite prototype by calculating the structural parameters using different exchange-correlations: PBEsol^17^, PBE^26,27^ and its correction due to Van der Waals interaction by means of semi-empirical Grimme’s DFT-D2^28,29^. Our results are summarized in Table 1. As expected, LDA underestimates and PBE overestimates the lattice parameters. Both PBEsol and van der Waal corrected PBE-D2 show very good agreement with the experimental results with less than 0.2% error in the lattice parameters. These results are in good agreement with DFT calculations done by Tunega *et al*. We thus choose PBESol for all the new chemically modified compounds^30^. In addition to the lattice constants, we also calculated the $\Gamma$-point phonon frequencies for lizardite using finite-displacement method as implemented in AFLOW*π*. The 51 normal of vibration are listed in Table 4 and are plotted in Fig. 2 together with the experimental results published by Balan *et al*.^7^ and are in reasonably good agreement with the experimental values.

**Table 1:** Optimized and experimental unit cell parameters (Å) and volumes (Å^3^) for lizardite, Mg_3_(Si_2_O_5_)(OH)_4_, with different functionals.

Functional	*a* = *b*	*c*	vol (Å^3^)
Exp^a^	5.332	7.233	178.086
PBEsol^b^	5.320	7.223	177.06
PBEsol	5.324	7.239	177.716
PBE	5.373	7.441	186.068
PBE-D2	5.324	7.243	177.826
LDA	5.244	7.052	167.934

The calculated and experimental angles are *α*, $\beta =90^\circ$ and $\gamma =120^\circ$ for all functionals.

^a^X-ray measurements^2^. ^b^Calculated with DFT by Tunega *et al*.^30^.

### Chemical Stability and Geometry

The new compounds (A_3_(Si_2_O_5_)(OH)_4_) are simulated by replacing the octahedral cation, Mg, in lizardite by one of 12 different elements which are isovalent to Mg^2+^; (A = Be, Ca, Ni, Mn, Fe, Zn, Al, Sn, Ge, Te, S, Se and Te). First, the chemical stability of the new compounds is investigated by calculating the theoretical formation energy (*E*_*f*_) given by

4 $${E}_{f}=E({{\rm{A}}}_{{\rm{Mg}}})-3{E}_{{\rm{A}}}-2{E}_{{\rm{Si}}}-4.5{E}_{{{\rm{O}}}_{2}}-2{E}_{{{\rm{H}}}_{2}},$$

where $E({{\rm{A}}}_{{\rm{Mg}}})$ is the total energy of the new structure with element A replacing the Mg position in lizardite chemical structure and *E*_X_ is the energy of the constituent X (A, Si, O_2_ and H_2_). The reference energies for calculating the chemical potential of the constituent elements are the bulk solids. For the prototype lizardite our calculated formation energy is −6154.94 kJ/mol in the same range as reported by a few experiments^11,31^. All the new compounds have a negative formation energy suggesting chemical stability. Compared to the prototype, E_*f*_ decreases for Be, Ca and is higher for the transition metal ion substitutions Mn, Fe, Zn and Ni. For verifying if the compound is indeed stable, we also analyze the geometry and elastic properties to screen for elements which show both mechanical and chemical stability.

The lattice parameters and the interatomic distances for all the twelve A_3_(Si_2_O_5_)(OH)_4_ relaxed systems are computed. Since we substitute the atom in the octahedral site (see Fig. 1), we look at the distortions in the cage. We find that only five substitutions (Ca, Mn, Ni, Fe, and Zn) preserve the octahedral structure. Furthermore, in case of Al, Sn, Ge, Te, S, Se and Be our computed values of the elastic properties show that one or more eigenvalues of elastic tensor is negative, thus contradicting Drucker’s stability criterion described in the Section 1. In the rest of the paper, we focus on the elements that fulfill the criteria of chemical, geometric and elastic stability: Ca, Mn, Fe, Ni and Zn. The values of the lattice constants and elastic constants for these cases are presented in Tables 2 and 3.

**Table 2:** Optimized and experimental unit cell parameters (Å) and bond lengths (Å) of lizardite and its chemical substitutions (A_3_(Si_2_O_5_)(OH)_4_), separated by group.

A	*a* = *b*	*c*	vol (Å^3^)	Si-O*a*	Si-Ob	A-OHin	A-OHout	A-O*a*	OHin	OHout
Mg^a^	5.332	7.233	178.086	1.616	1.646	2.083	2.021	2.121	0.794	0.837
Mg	5.324	7.239	177.716	1.599	1.659	2.087	2.021	2.142	0.974	0.979
Ca	5.642	7.519	207.282	1.598	1.720	2.282	2.229	2.283	0.981	0.974
Mn	5.433	7.351	187.940	1.602	1.680	2.151	2.099	2.217	0.979	0.985
Fe	5.247	7.296	173.962	1.602	1.653	2.081	2.170	2.196	0.982	0.985
Ni	5.281	7.174	173.266	1.602	1.656	2.043	2.002	2.101	0.979	0.985
Zn	5.355	7.245	179.933	1.598	1.667	2.091	2.035	2.172	0.977	0.984

The letter A is the element substituted in the lizardite structure. The calculated and experimental angles are $\alpha =\beta =90^\circ$ and $\gamma =120^\circ$ for all systems.

^a^Mellini^2^.

**Table 3:** Calculated bulk (*B*), shear (*G*) moduli for Voigt (*V*) and Reuss (*R*) averaging procedure, Young’s modulus (*E*) and Poisson’s ratio ($\nu$), elastic constants and the universal elastic anisotropy index (A^*U*^) of lizardite and its chemical substitutions (A_3_(Si_2_O_5_)(OH)_4_), separated by group.

A	*B*_*V*_	*B*_*R*_	*G*_*V*_	*G*_*R*_	*E*_*H*_	*ν*_*H*_	*c*_11_	*c*_12_	*c*_13_	*c*_14_	*c*_33_	*c*_44_	*c*_66_	A^*U*^
Mg^a^	95.66	80.69	53.85	36.23	115.46	0.28	235.61	85.96	25.05	2.69	118.16	20.92	74.83	2.62
Mg	82.45	62.47	47.21	26.52	95.56	0.28	217.2	76.2	17.0	0.2	87.0	14.3	70.5	4.22
Ca	69.57	55.56	34.99	20.92	72.99	0.31	167.8	65.9	20.9	0.4	75.2	11.4	51.0	3.62
Mn	79.11	62.07	38.45	24.71	82.45	0.31	187.6	80.0	23.5	0.2	82.8	14.0	53.8	3.05
Fe	80.17	69.70	42.62	27.53	91.02	0.30	190.4	69.8	24.4	0.1	103.7	15.4	60.3	2.89
Ni	81.18	65.31	43.50	27.64	91.84	0.29	199.8	71.9	24.6	0.2	88.8	15.6	63.9	3.11
Zn	85.62	65.27	42.04	26.45	89.23	0.30	206.8	90.1	22.4	0.0	87.4	14.9	58.3	3.26

The letter A is the element substituted in the lizardite structure. All data are given in GPa, except *ν*_*H*_ which is dimensionless.

^a^Calculated by Mookherjee and Stixrude^9^.

**Table 4:** Calculated vibrational frequencies for lizardite at $\Gamma$ point.

Mode	Wavenumber (cm^−1^)	Mode	Wavenumber (cm^−1^)	Mode	Wavenumber (cm^−1^)
4	120.59	22–23	401.31	40–41	710.36
5–6	125.20	24–25	421.03	42	722.42
7–8	208.92	26–27	434.97	43–44	780.06
9	224.53	28–29	453.97	45*	784.66
10–11	268.65	30*	464.61	46–47	909.71
12–13	294.27	31	479.34	48*	954.45
14–15	310.53	32–33	484.79	49*	1025.27
16*	325.24	34	528.48	50	1039.54
17	342.98	35–36	588.60	51–52	3725.66
18–19	357.60	37–38	650.05	53	3747.31
20*	371.54	39	666.49	54	3834.30
21	379.92				

The simbol (*) indicates the IR silent modes.

The variation in the lattice parameter for the elements is consistent with the ionic radius^32^ of the substituting element with the exception of Mn. The Mn^2+^ ionic radius is smaller, 0.83 Å compared to 0.89 Å in Mg^2+^, which should reduce the volume of the cell but we find an increase in both *a* and *c* by 2%. The largest change is observed for Ca_3_(Si_2_O_5_)(OH)_4_, consistent with the larger size of the Ca atom compared to Mg. In all the five new compounds, the tetrahedral and (OH)^−^ bonds lengths are almost unchanged. Our results of geometry for Ni_3_(Si_2_O_5_)(OH)_4_ are in good agreement with experiments of synthesized Ni-substituted for Mg_3_(Si_2_O_5_)(OH)_4_-nanotubes by hydrothermal reactions^33^.

The total charge on the substituted transition metal (TM) atoms in the new compounds can be obtained from the Lowdin’s charge. We observed that in all structures the net charge on the substituted element is 7.0 in the prototype, 6.7 in Ca, 6.5 in Mn, 6.5 in Fe, 6.4 in Ni and 6.6 in Zn new composites. The charges in Si and H elements are almost the same as before the chemical substitution. After relaxation, the structures replaced with Mn, Fe and Ni show a magnetic moment of 4.0 *μ*_*B*_/atom, 3.4 *μ*_*B*_/atom and 1.6 *μ*_*B*_/atom in the unit cell, respectively.

### Elastic Constants and Anisotropy

**Figure 3:**
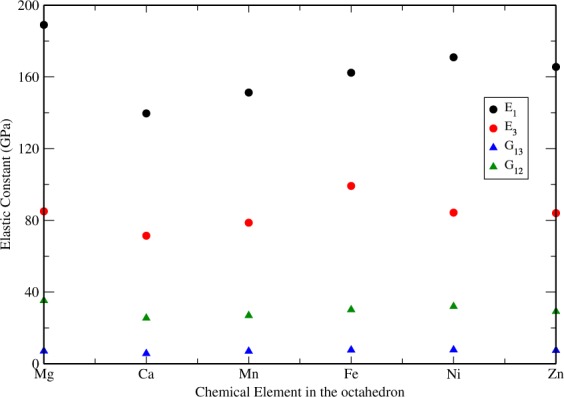
Elastic constant for new compounds formed by chemical substitutions: *E*_*i*_, the Young’s modulus in direction *i* and *G*_*ij*_, the shear stiffness for shearing in the *i*–*k* plane.

Elastic constants are fundamental properties of materials and can be used to judge the mechanical stability with different chemical compositions or crystal structures. In this work we compute the full elastic tensor for the five chemically modified lizardite compounds. In Table 3, we present our calculated results of the averaged bulk modulus *B*, shear modulus *G*, Young’s modulus *E* and Poisson’s ratio ($\nu$). Figure 3 shows the Young’s modulus in direction 1 and 2 as well as the shear stiffness *G*_12_ and *G*_13_. The calculated universal anisotropic index *A*^*U*^ is also given which is a measure of the anisotropic degree of the crystal as described in Sec. 1. For the prototype, our computed value of bulk modulus $B=72.46\,{\rm{GPa}}$ and the experimental values presented in the literature are single crystal X-ray diffraction studies (XRD) ($B=57\,{\rm{GPa}}$)^3^, shock wave equation of state ($B=(63.5\pm 3.5)\,{\rm{GPa}}$ at low pressure)^5^ and synchrotron X-ray diffraction applying to P-V equations of state ($B=71.0(19)\,{\rm{GPa}}$ for volume = 180.92 Å^3^)^6^. Both *B* and *G* do not vary much with chemical modifications. The value of Poisson’s ratio, $\nu$, which is a measure of the ductility/brittleness of materials varies from 0.28 in Mg to 0.31 in Mn, indicating that the prototype and all the derived new compounds are more brittle, in accordance with Frantsevich’s criteria^34^.

One important mechanical property for modeling correctly rock and fluid processes is the *anisotropy of elasticity* which is determined by computing the full elastic tensor, as discussed below. As shown in Table 5, the ratio of ${c}_{11}/{c}_{33}=2.50$ which means that in-plane stiffness is much larger than the out-of-plane c-axis, under a uniaxial stress. Also, ${c}_{66}/{c}_{44}=4.93$ shows that the basal plane is more resistant to fracture (rigidity) than c-axis when a shear stress is applied. These values are in good agreement with experimental results^6^ and indicate strong anisotropic materials. Our results are different from other DFT calculations with LDA pseudopotentials done by Mookherjee and Stixrude^9^ (20% for *c*_11_/*c*_33_ and 27.4% for *c*_66_/*c*_44_) and atomistic simulations presented by Auzende *et al*.^10^ (100% for *c*_11_/*c*_33_ and 11.4% for *c*_66_/*c*_44_). The difference arises from the choice of the LDA functional which underestimates the lattice constants and consequently affects the elastic constants.

**Table 5:** Calculated density ($\rho$ in 10^3^kg/m^3^), Hill-averaged bulk (*B*) and shear (*G*) moduli (in GPa), Pugh’s index (*B*/*G*), elastic constant ratio *c*_*ii*_/*c*_*jj*_, compressional, *V*_*p*_, and stress, *V*_*s*_, wave velocities (in m/s) of lizardite and its chemical substitutions (A_3_(Si_2_O_5_)(OH)_4_), separated by group.

A	*ρ*	*B*	*G*	*B*/*G*	*c*_11_/*c*_33_	*c*_66_/*c*_44_	*V*_*p*_	*V*_*s*_	*V*_*p*_/*V*_*s*_
Mg^a^	—	88.18	45.04	1.96	1.99	3.58	6360	3630	1.75
Mg	2.589	72.46	36.87	1.97	2.50	4.93	6854	3773	1.81
Ca	2.599	62.57	27.96	2.24	2.23	4.47	6198	3280	1.88
Mn	3.260	70.59	31.58	2.24	2.27	3.84	5879	3112	1.88
Fe	3.548	74.94	35.08	2.14	1.84	3.92	5857	3144	1.85
Ni	3.644	73.25	35.57	2.06	2.25	4.10	5754	3124	1.84
Zn	3.695	75.45	34.25	2.20	2.37	3.91	5725	3044	1.88

The letter A is the element substituted in the lizardite structure.

^a^Mookherjee and Stixrude^9^ with DFT.

Additionally, the anisotropy factor can also be estimated from the compliance tensor, *s*_*ij*_, and the Young’s modulus. From our calculated elastic constants, ${s}_{33}/{s}_{11}={E}_{1}/{E}_{3}=2.22$, the ratio between the Young’s moduli is not the same. The lizardite anisotropy can also be seen from linear compressibility, *β*_*i*_, defined as material’s response to decrease in length of a line when the crystal is subject to hydrostatic pressure in direction $i=1,2,3$. From our lizardite results, ${\beta }_{1}={s}_{11}+{s}_{22}+{s}_{33}=0.00281\,{\rm{GPa}}$, ${\beta }_{3}=2{s}_{13}+{s}_{33}=0.01040\,{\rm{Gpa}}$ and ${\beta }_{3}/{\beta }_{1}=3.70$. We obtain good results compared to experimental ones that reported ${\beta }_{1}=0.002735\,{\rm{GPa}}$, ${\beta }_{3}=0.009707\,{\rm{GPa}}$ and ${\beta }_{3}/{\beta }_{1}=3.55$^6^. Poisson’s ratio of lizardite-chrysotile serpentinites average is 0.36 at 1 GPa^1^ and our result is 0.28 GPa at zero pressure. High anisotropy of the lizardite is also shown in the Poisson’s ratio, ${\nu }_{ij}$, that describes the response in the direction orthogonal *j* to this uniaxial stress *i*. We report for lizardite ${\nu }_{12}=-\,{s}_{21}/{s}_{11}=0.34$, ${\nu }_{13}=-\,{s}_{31}/{s}_{11}=0.13$, ${\nu }_{31}=-\,{s}_{13}/{s}_{33}=0.06$. Finally, we also compute the universal anisotropy index parameter A^*U*^ as described in Sec. 1. For the prototype composition, the value is around 4.22 which is higher than most inorganic compounds^23^ and is further proof of strong crystal anisotropy in this mineral class. Our computational approach describes all the elastic properties for the prototype lizardite in good agreement with experiments and provides references to analyze the new isostructural compounds. Compared to the prototype, *A*^*U*^ decreases with the chemical substitutions, with Fe-lizardite being the least anisotropic among the list (see Table 3). The relations between the compressional elastic constants ${c}_{11} > {c}_{33}$ and the shear elastic constants ${c}_{66} > {c}_{44}$, shown in Table 5, are lower compared to lizardite, suggesting higher stiffness and lower rigidity in the new compounds.

In Table 5, we tabulate the Hill-averaged shear (*G*) moduli, bulk (*B*) moduli and Pugh’s indexes ($B/G$). The smaller *G* values for the five compounds indicate low resistance to stress deformations. For Fe, Ni and Zn, *B* is slightly higher which means these materials have high fracture strengths. The higher Pugh’s indices ($B/G$) in all the new compounds, suggest that they are more ductile than the prototype lizardite.

One other parameter that can be extracted from our first principles elastic constants is the ratio of compressional (*V*_*p*_) to shear wave velocity (*V*_*s*_). This information is often used as a lithology indicator and is important for petrophysical evaluation, seismic imaging and modeling geomechanical properties. We calculated the compressional, *V*_*p*_, and shear, *V*_*s*_, wave velocities using Eq. (2) and the results are presented in Table 5, together with the DFT results obtained by Mookherjee and Stixrude^9^ and with atomistic simultations reported by Auzende *et al*.^10^. Our calculated values for the ideal protoype lizardite composition lies in the range of experimental values ranging from 5.03 to 6.33 km/s for *V*_*p*_ and 2.62 to 3.38 km/s for *V*_*s*_ as reported for three different samples by Watanabe *et al*.^35^. With chemical variations, we see a decrease in two velocities but the ratio increases. The difference between the compressional velocities of waves in lizardite and the new compounds is around 1 km/s and between shear velocities is around 0.7 km/s, therefore, they should be detectable in practice.

## Conclusions

We used *ab initio* DFT to study changes in the physical properties of the clay mineral lizardite (Mg_3_(Si_2_O_5_)(OH)_4_) due to substitutions of the metal cation, Mg, in its octahedral sheet, by 12 different atoms from different chemical groups. We compute the formation energies, optimal lattice parameters, elastic constants and acoustic velocities. Of the twelve substitutions, Be, Te, Sn, Al, S, Se and Ge had negative eigenvalues in elastic tensor not satisfying Drucker’s criteria along with the *A*_*U*_ smaller than one. Only five elements, Ca, Mn, Fe, Ni and Zn survived the chemical and mechanical criteria. Overall, in this work, we show that the new compounds are less resistant to mechanical deformations, more malleable and less anisotropic compared to the prototype lizardite.
